# A curated dataset of peste des petits ruminants virus sequences for molecular epidemiological analyses

**DOI:** 10.1371/journal.pone.0263616

**Published:** 2022-02-10

**Authors:** Michael D. Baron, Arnaud Bataille

**Affiliations:** 1 The Pirbright Institute, Pirbright, Surrey, United Kingdom; 2 CIRAD, UMR, ASTRE, Montpellier, France; 3 ASTRE, University of Montpellier, CIRAD, INRAE, Montpellier, France; University of Reunion Island, RÉUNION

## Abstract

Peste des petits ruminants (PPR) is a highly contagious and devastating viral disease infecting predominantly sheep and goats. Tracking outbreaks of disease and analysing the movement of the virus often involves sequencing part or all of the genome and comparing the sequence obtained with sequences from other outbreaks, obtained from the public databases. However, there are a very large number (>1800) of PPRV sequences in the databases, a large majority of them relatively short, and not always well-documented. There is also a strong bias in the composition of the dataset, with countries with good sequencing capabilities (e.g. China, India, Turkey) being overrepresented, and most sequences coming from isolates in the last 20 years. In order to facilitate future analyses, we have prepared sets of PPRV sequences, sets which have been filtered for sequencing errors and unnecessary duplicates, and for which date and location information has been obtained, either from the database entry or from other published sources. These sequence datasets are freely available for download, and include smaller datasets which maximise phylogenetic information from the minimum number of sequences, and which will be useful for simple lineage identification. Their utility is illustrated by uploading the data to the MicroReact platform to allow simultaneous viewing of lineage date and geographic information on all the viruses for which we have information. While preparing these datasets, we identified a significant number of public database entries which contain clear errors, and propose guidelines on checking new sequences and completing metadata before submission.

## Introduction

Peste des petits ruminants (PPR) is a highly contagious, high mortality, disease affecting predominantly sheep and goats, and caused by a virus of the genus *Morbillivirus* in the family *Paramyxoviridae* [1]. The name of the PPR causative agent remains peste des petits ruminants virus, with abbreviation PPRV, although the International Committee on Taxonomy of Viruses (ICTV) recently changed its species name to *Small ruminant morbillivirus* [2]. This purely taxonomic change seems to have caused confusion in experts and non-experts alike, with the name small ruminant morbillivirus, and abbreviation SRMV, appearing in the scientific literature and PPRV sequence submissions to GenBank as the name of the virus, rather than its species.

PPRV is a non-segmented, negative-sense single-stranded RNA virus. The genome of approximately 16kb in length contains 6 genes encoding 6 structural proteins (the nucleocapsid protein (N), the phosphoprotein (P), the matrix protein (M), the fusion protein (F), the haemagglutinin protein (H) and the polymerase protein (L)) plus two non-structural proteins (C and V) encoded in alternative open reading frames in the P gene [1]. Most evolutionary studies of PPRV have been based on partial gene sequences, typically the short (250-350bp) portions of the N and/or F genes that are produced by either of the original PPRV-specific RT-PCRs developed for diagnostic use [3, 4]. Such studies have identified four distinct genetic lineages (I-IV) of PPRV [5] with shifting geographic distribution [6–8], and these relatively short sequences remain the main type of PPRV sequence in the database, although the number of full gene and full genome sequences of PPRV strains has increased in the last few years, allowing for more robust and more complex phylogenetic analyses [9–11].

PPR represents a significant threat to food security and to the livelihoods of smallholder farmers, particularly in the developing world, and is now the target of a global eradication campaign led by the OIE and FAO [12]. The success of this effort will depend on robust and rigorous research to improve our understanding of this pathogen [13] as well as good disease surveillance and reporting [14]. As part of the contribution to the latter, the OIE has recently commissioned the establishment of a network of diagnostic laboratories carrying out significant work on PPRV [15]. For the benefit of such laboratories, the designated OIE Reference Laboratories for PPR are setting up a website containing useful protocols and information on PPRV, including the latest phylogenetic and phylogeographic information. We have prepared sets of PPRV sequences for such analyses, including both comprehensive sets of known sequences and minimal sets containing all the information needed for lineage and subclade identification of novel sequences but without duplicates or biases towards sequences from any one clade. While preparing these datasets, we found a number of problems in the available sequences in the publicly available databases (GenBank/EMBL), problems both with the metadata provided in the database submissions and with the quality of some of the PPRV sequences, including problems with some of the full-length PPRV genomes published. These problems may have influenced the results and conclusions of previous phylogenetic studies on PPRV.

## Materials and methods

### Sequence acquisition

All PPRV/SMRV sequences in GenBank were acquired in April 2021 as an XML file. A custom script was used to extract the accession number, the sequence and other metadata (gene/genome, country, location, collection date, host, isolate and strain) for each database entry. Isolate location and date information, where not given or given in minimal detail in the GenBank database, was supplemented by literature search to find any published information available, and this was added to the dataset. Further sorting was carried out in Excel.

### Sequence analysis

Sequence alignments were prepared using MAFFT [16] and viewed/edited using AliView [17]. Alignment shading was carried out with pyBoxshade [18]. Maximum likelihood phylogenetic trees were prepared using RAxML [19] using the GTRGAMMA model of nucleic acid evolution and 20 independent tree searches, or IQ-TREE 2 [20]. Branch support for clades was calculated using the Shimodaira-Hasegawa-like modified approximate likelihood ratio test (SH-aLRT) [21] as implemented in IQ-TREE 2. Values greater than 80% for SH-aLRT were taken to indicate strong support [22]. The resultant unrooted trees were viewed and figures prepared using FigTree; for ease of presentation, pseudo-rooted trees are presented, with the root placed at the midpoint.

Divergence filtering was carried out with TreeTime [23] using a clock-filter setting of 3.5, i.e., we exclude sequences which diverge from the best-fit clock by more than 3.5 x the inter-quartile range for all such divergences. Maximum likelihood trees calculated after removing vaccine and vaccine-like sequences, and lineage-specific subtrees were analysed by TreeTime using the branch lengths of the input trees.

Screening for mixed sequences was carried out using the tests RDP, GENECONV, Bootscan, Maxchi, Chimaera, SiScan and 3Seq as implemented in RDP5 (see [24] and references therein). For Bootscan and SiScan tests, a window size of 200 and a step size of 50 were set, otherwise the default settings for each test was used. A probability cut off value of 0.05, after Bonferroni correction, was set, and only events identified by 4 or more tests were considered. Fractional identity values for pairs of sequences were calculated using the Recombination Analysis Tool program [25], with a window size of 200 and a step size of 100.

Sequence duplicates and subsequences were identified using a custom Python program, available from the authors by request. Dataset pruning was performed using *treemmer* [26], pruning the shortest branch from the closest pair at each step.

### Sequence naming

It was quickly apparent when reviewing all the PPRV sequences in the database that there is no consensus as to how virus isolates should be named. We have adopted a naming convention based on that used for influenza virus, where the virus is named for the place and date of its isolation, with the place being broken into country and subdivision within the country, to the level of the smallest identifiable such subdivision recorded (e.g. village, town, county, province), e.g. PPRV/Pakistan/Punjab/Okara or PPRV/Tanzania/Mtwara/Tandahimba. For simplicity in this paper we have restricted isolate names to the country and smallest identified location unit in the country, and restricted the date annotation to just the year (many sequences in any event do not record any more detail than the year). We have also left out the initial virus identifier, as all the sequences in our analyses are PPRV. Most PPRV isolates are from domestic small ruminants (sheep or goats): we adopted a similar convention to that used for influenza virus isolates, where the host is only given if it is not human, and only added the host where the isolate is from a host other than domestic sheep or goats, e.g. KT633939, isolated from an ibex in Bazhou, China, in January 2015, for example, becomes China/Bazhou/2015/ibex. Where there was more than one isolate from the same place and time, we added unique identifiers used by the original authors, e.g. database entries KF479408 to KF479419 become Nigeria/Taraba/2012/1 to Nigeria/Taraba/2012/19 and, since we are naming sequences from the isolates, rather than the isolates themselves, and since some isolates are represented by multiple sequences in the database, we prepended the accession number as part of the sequence name. Vaccines were not assigned to a specific country, since they have been passaged in cell culture > 50 times, and therefore will have diverged a lot from the original sequence representative of that place and time. Instead, known vaccine viruses were labelled “vaccine/common-name-of-vaccine”, e.g. vaccine/Nigeria75, vaccine/Sungri96.

## Results

### Sequence acquisition

A total of 1886 sequences were extracted from the database. Patents (5 entries) and primer sequences (50 entries) were removed, as were sequences that had no identifiable date (5 entries) and any remaining sequences that were less than 200 bases in length (14 entries). Two entries (EF641264 and EF641263) were from an unnamed strain that had been experimentally put into, and subsequently isolated from, cattle in India, but were otherwise unidentifiable. This left 1810 partial gene sequences, full gene sequences or complete genome sequences. These database entries were then screened for quality and to eliminate replicate sequences of the same isolate. A graphical overview of the screening and filtering workflow is presented in S1 Fig.

### Complete genome sequences

Several problems were apparent in the alignment of the whole genome sequences. The most common region to show problems after aligning the genomes was the long untranslated region (UTR) between the M and F protein coding sequences, i.e. the M gene 3’ UTR and the F gene 5’ UTR. Together these form an UTR of >1kb that has a very high GC content. This high GC content can cause problems when using Sanger sequencing unless the reactions are modified [27]. Poor coverage of this region has also been reported, for PPRV and rinderpest virus, when using next generation sequencing technologies such as Illumina [28, 29]. Visual inspection of the available full genomes showed that there were many errors in this region, such as two extra copies of a 52-base sequence, making the genome 104 bases longer than normal (KM816619), or extra gaps required to align the sequence with other genomes (most) (see S2 Fig). Two genomes (KY967609 and KY967610) lacked the CTT motif at the M-F gene boundary, even though this motif is absolutely conserved across all morbilliviruses. All of these problems were in addition to the known gain of six bases in this region by a clade of PPRV that appeared in China in 2013 [30]. In many cases, the alignment strongly suggested that bases had been lost in some positions and gained in others in an apparently random fashion in many virus isolates. While it is, in theory, possible that there are naturally occurring deletions and insertions in these viruses, such naturally occurring genome modifications would have been maintained in the population and observed in other isolates, as was found for the 6 base insertion identified in China [11]. However, the small insertions and deletions observable when all available genome sequences were aligned were always unique to one database entry, suggesting that this sequence variation is actually a result of the known difficulty in obtaining accurate sequence in this region. In order to avoid distortion of downstream analyses by apparent sequence divergence that is actually sequencing error, we removed the whole M-F UTR from the full genome alignments, and recommend that this region not be included in other analyses as the available data are inherently unreliable.

Other clear errors in the full genome sequences deposited in the database were the inclusion in the genome of 1 or 3 extra G residues at the P gene editing site [31] (seen in KP868655 and KM089831), presumably because the sequence at that point was derived from edited mRNA transcripts rather than the viral genome or anti-genome RNA, and a 6 base insertion in the antigenome promoter (seen in MN369542), a promoter that is otherwise completely conserved across all PPRV genomes. A full list of these genome sequence corrections is given in S1 Table.

The full set of genomes (minus the M-F UTR, making the length 14898) was then analysed with RDP5 to look for apparent recombination. Actual recombination can occur in the paramyxoviruses during co-infection with two strains of the same virus [32–35], although it appears to be rare in nature [36–38]. On the other hand, many full PPRV genome sequences are obtained by sequencing (whether by standard Sanger sequencing or next-generation sequencing) of separately-obtained PCR products. It is possible for one or more of those PCRs to preferentially amplify contaminating RNA from another PPRV strain present in the laboratory, giving rise to a genome sequence that is actually a mixture of sequences from two or more viruses, and so appears to be recombination [38]. Whatever the cause, it is important to identify mixed genome sequences in the dataset, as such sequences will distort and undermine phylogenetic analyses [39, 40]. Several genomes were identified by RDP5 as potentially mixtures of sequence from more than one genome and are listed in Table 1.

**Table 1:** Results of RDP5 analysis of available PPRV genome sequences. The table shows the genome sequences for which RDP5 identified a recombination signal in at least 4 out of 7 tests, the genome identified as the most likely (of the available sequences) contributor of contaminating sequences, the region of the genome affected and the RDP5 tests which gave a positive signal.

Suspect genome	Contaminant	Begin	End	RDP5 tests positive^1^
KT633939/China/Bazhou/2015	KY628761/vaccine/Nigeria75	5560	5860	RGBMCST
KT633939/China/Bazhou/2015	KY628761/vaccine/Nigeria75	8021	8492	RGBMCST
KR261605/India/Gingee/2014/2	KJ867542/vaccine/Sungri96	9368	9858	RGB––ST
KJ867543/Uganda/Kotido/2012	KC594074/Morocco/2008	4110	4488	–GBMCS–
KF727981/vaccine/Sungri96	AJ849636/Turkey/Sakarya/2000^2^	1	4096	RGBMCST
KF727981/vaccine/Sungri96	AJ849636/Turkey/Sakarya/2000^2^	9991	14898	RGBMCST
KR828814/Nigeria/Kwara/2012	MN657232/Turkey/Central_Anatolia/2018	566	1049	RGBMC–T
KR828814/Nigeria/Kwara/2012	KR828813/Nigeria/Yobe/2013	3096	4001	RGBMCST
KR828814/Nigeria/Kwara/2012	KR828813/Nigeria/Yobe/2013	5326	5785	RGBMCS–
KY967608/Pakistan/Lahore/2015	KT860065/India/Tamil_Nadu/2015/4^3^	3070	5559	RGBMCST
KY967609/Pakistan/Faisalabad/2015	KP260624/China/2014/BJ^4^	8214	8840	RGBMCST
KY967610/Pakistan/Layyah/2015	KP260624/China/2014/BJ^4^	8214	8840	RGBMCST

^1^The letter code indicates in which of the tests implemented in RDP5 a statistically significant indication of “recombination” was found after Bonferroni correction for multiple tests: R = RDP; G = GENECONV; B = Bootscan; M = Maxchi; C = Chimaera; S = SiScan; T = 3Seq.

^2^Or other Turkish isolate for which the full genome is not available.

^3^Or any one of 8 related genomes from India or the UAE.

^4^Or any one of 27 closely related genomes from China.

**Fig 1:**
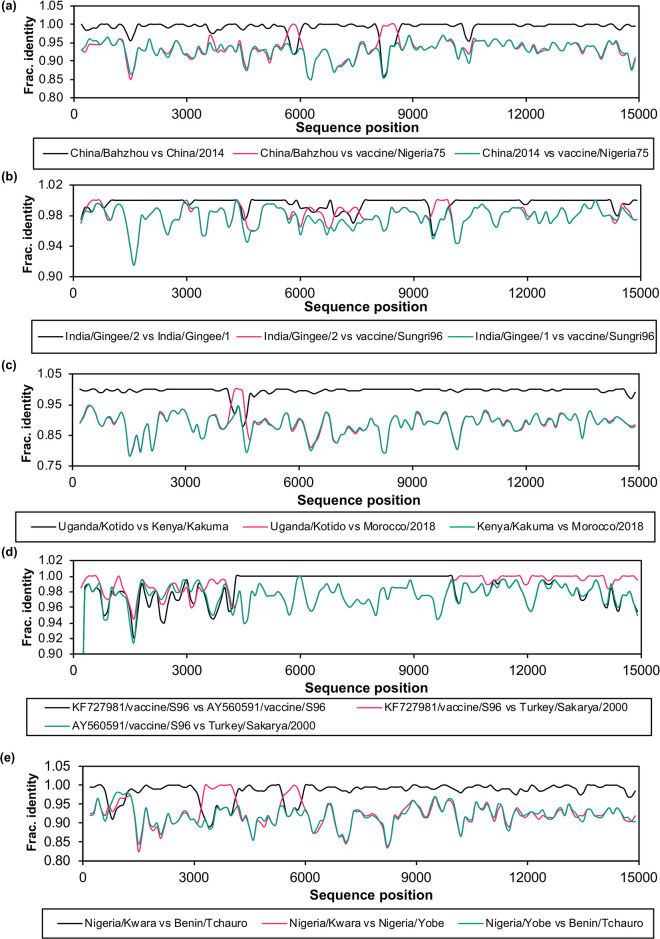
Analysis of possible contaminating sequences in PPRV genome sequences. The fractional identity (Frac. Identity) was calculated as described in Methods for the pairs of sequences indicated in the legend to each graph, these being, in each case, the suspect sequence vs the suggested major parent, the suspect sequence vs the suggested minor parent, and the major parent vs the minor parent. The suspect sequences were (a) KT633939/China/Bazhou/2015; (b) KR261605/India/Gingee/2014/2; (c) KJ867543/Uganda/Kotido/2012; (d) KF727981/vaccine/Sungri96; (e) KR828814/Nigeria/Kwara/2012.

We further investigated these cases by looking at the level of sequence identity between the suspect genome and the genomes identified by RDP5 as the major and minor parent, i.e. the most similar genome for most of the length of the genome, and the suspected contaminant (Fig 1).

These data provided a strong rationale for removing KF727981, KJ867543, KT633939, KR261605 and KR828814 from our dataset as they are clearly natural or artefactual recombinants.

**Fig 2:**
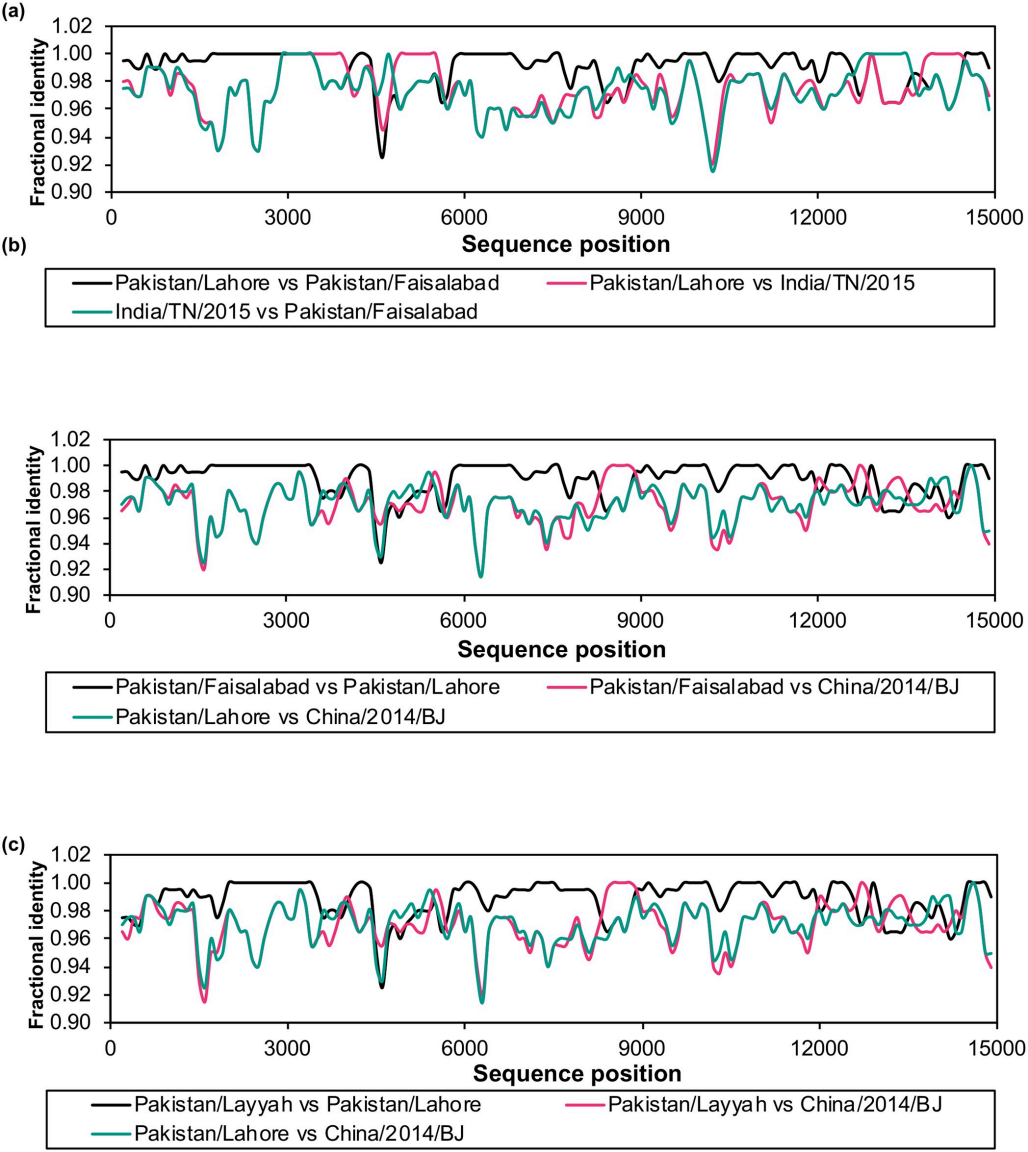
Analysis of possible contaminating sequences in genome sequences for PPRV isolated in 2015 in Pakistan. The fractional identity was calculated as described in Methods for the indicated pairs of sequences, these being, in each case, the suspect sequence vs the suggested major parent, the suspect sequence vs the suggested minor parent, and the major parent vs the minor parent. The suspect sequences were (a) KY967608/Pakistan/Lahore/2015; (b) KY967609/Pakistan/Faisalabad/2015; (c) KY967610/Pakistan/Layyah/2015.

RDP5 also gave strong indications of recombination in three genomes from Pakistan, KY967608/Pakistan/Lahore/2015, KY967609/Pakistan/Faisalabad/2015 and KY967610/Pakistan/Layyah/2015. However, unlike in the cases above where the main sequences and the potential contaminants are geographically and/or temporally distinct, the three Pakistani genomes and the suggested contaminants (from China in 2014, or India in 2014–2015) are all closely related, with very small differences in sequence (Fig 2). As the sequences are so closely related, it is possible to find multiple regions where the suggested minor parent/contaminant is identical to the suspect sequence, and sometimes also to the suggested major parent (Fig 2). In the absence of corroborating data, therefore, we have retained these three genome sequences.

### Sequences of individual genes

The remaining sequences were then sorted by gene. There are too few P and L gene sequences available (4 and 7, respectively, not counting genome sequences) to use for any useful analysis, so those sequences were no longer considered. Data for the N, M, F and H genes were grouped separately, including the full length gene sequences for these genes available from the whole genome sequences. Because short partial N and F gene sequences have been used to characterise outbreak viruses for many years, the N and F gene sets consisted primarily of these short sequences, with only 171/1118 and 92/495, respectively, covering the full coding sequence, while the M and H gene datasets were 148/186 and 103/107 full length, respectively. The resultant alignments for the N and F genes therefore contain a high proportion of missing data, but including this is preferable to cropping the alignment to just the region covered by most of the sequences. All the main programs used for phylogenetic analysis of such alignments (e.g. RAxML, IQ-TREE 2, Mr Bayes, BEAST) deal with this missing data appropriately.

For the N and F datasets, since the primers used to generate the short sequences are known [4, 41], we identified those database entries that had retained part or all of the standard primer sequences and trimmed the published sequences to remove the primer sequences. The gene-specific datasets were then filtered to remove poor quality sequences and to identify database entries matching the sequence of one of the common vaccine viruses and therefore probably arising from laboratory contamination.

#### (i) Quality filtering

For each gene, the available sequences (including the respective regions from the complete genome sequences) were first aligned with MAFFT. Database entries that could not be aligned with the other PPRV sequences without introducing two or more gaps were excluded.

The sequences in each alignment were further checked for possible sequencing errors by looking for stop codons in the relevant open reading frame, and we excluded any sequence that had such a stop codon, plus two sequences that did not have a stop codon where they should have (MK213755, which encompasses the end of the N protein open reading frame, and KF992717, which encompasses the end of the H protein open reading frame, both lack the expected stop codon at the relevant point); the reasonable assumption here is that sequences that have not been checked by the original authors, or checked so badly that they missed a stop codon in the virus open reading frame, should be considered suspect. All of the cases of internal stop codons were found in the N gene sequences. A computer-readable text file, S1 List, contains a list of accession numbers of all sequences excluded on basic quality grounds.

#### (ii) Vaccine contamination

Maximum likelihood phylogenetic trees were then built for N, M, F and H gene sequences. The sequences clustering in the same clades as vaccine/Nigeria75 or vaccine/Sungri96 sequences were further examined to identify entries that arose from contamination.

**Fig 3:**
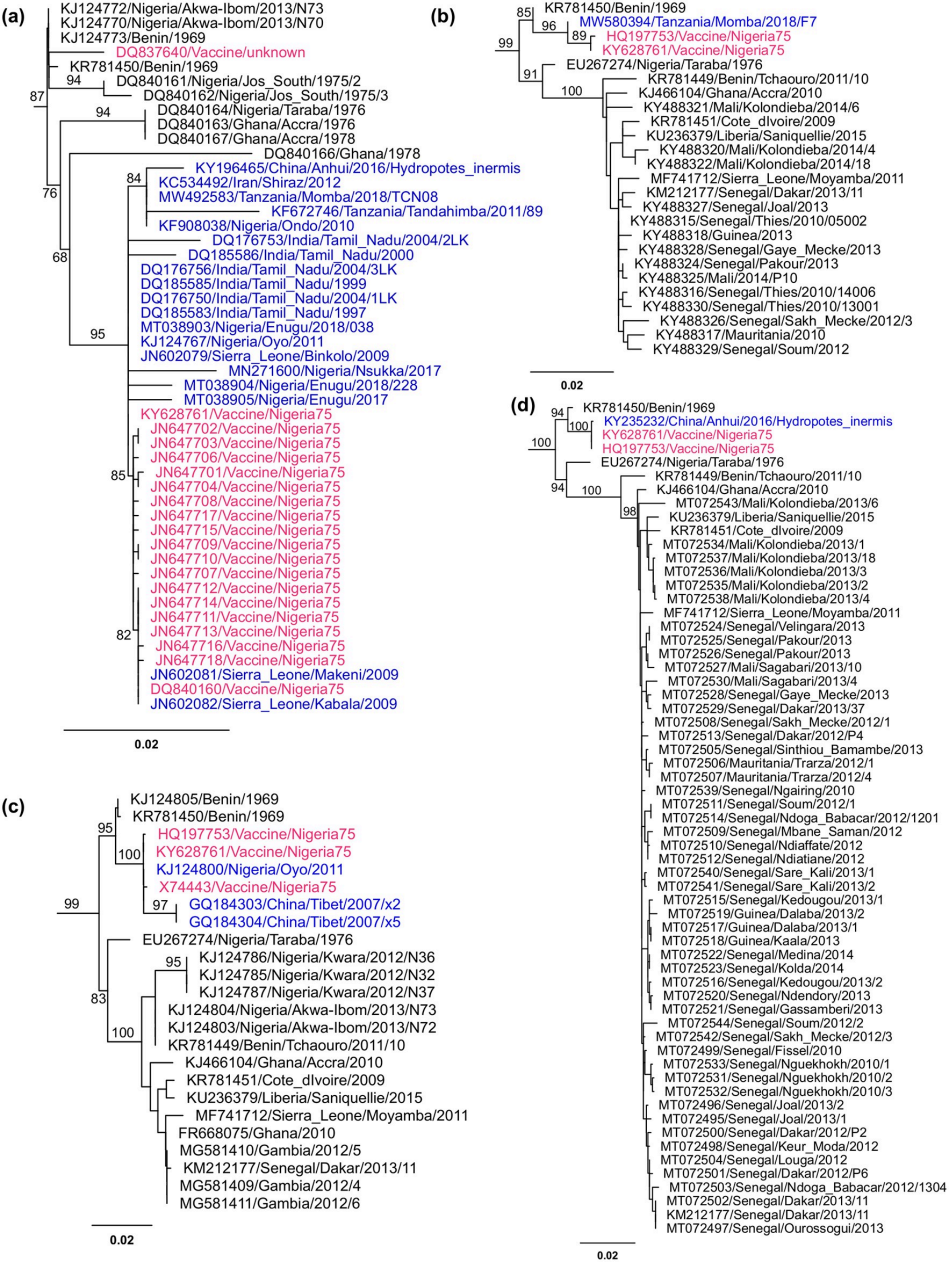
Identification of vaccine/Nigeria75 contaminants in the sequence database. Maximum likelihood trees were constructed in IQ-TREE 2 after quality filtering: (a) N gene sequences, (b) M gene sequences, (c) F gene sequences and (d) H gene sequences. From these trees either the part of the Lineage 2 clade containing vaccine/Nigeria75 sequences (a) or the whole Lineage 2 clade (b-d) were extracted and shown here. Branch support values shown are SH-aLRT values. Sequences shown in red are those listed in the database as being vaccine/Nigeria75, while those in blue are sequences that were eliminated as they should not have been similar to the vaccine/Nigeria75 sequence. The scale bar shows the branch length scale in units of substitutions per site.

Identifying database entries that are actually vaccine/Nigeria75 is relatively easy, as this vaccine is derived from a very early isolate and there are few sequences that would be expected to be similar; certainly a sequence supposedly from China or India, for example, but which is closely related to vaccine/Nigeria75, is clearly an error. One H gene sequence (KY235232) and one M gene sequence (MW580394) were found to be essentially identical to vaccine/Nigeria75 sequence. A total of 19 N gene sequences and 3 F gene sequences, from various laboratories, were similarly found to be the vaccine/Nigeria75 strain, and in all cases the relevant clade had strong statistical support (Fig 3).

**Fig 4:**
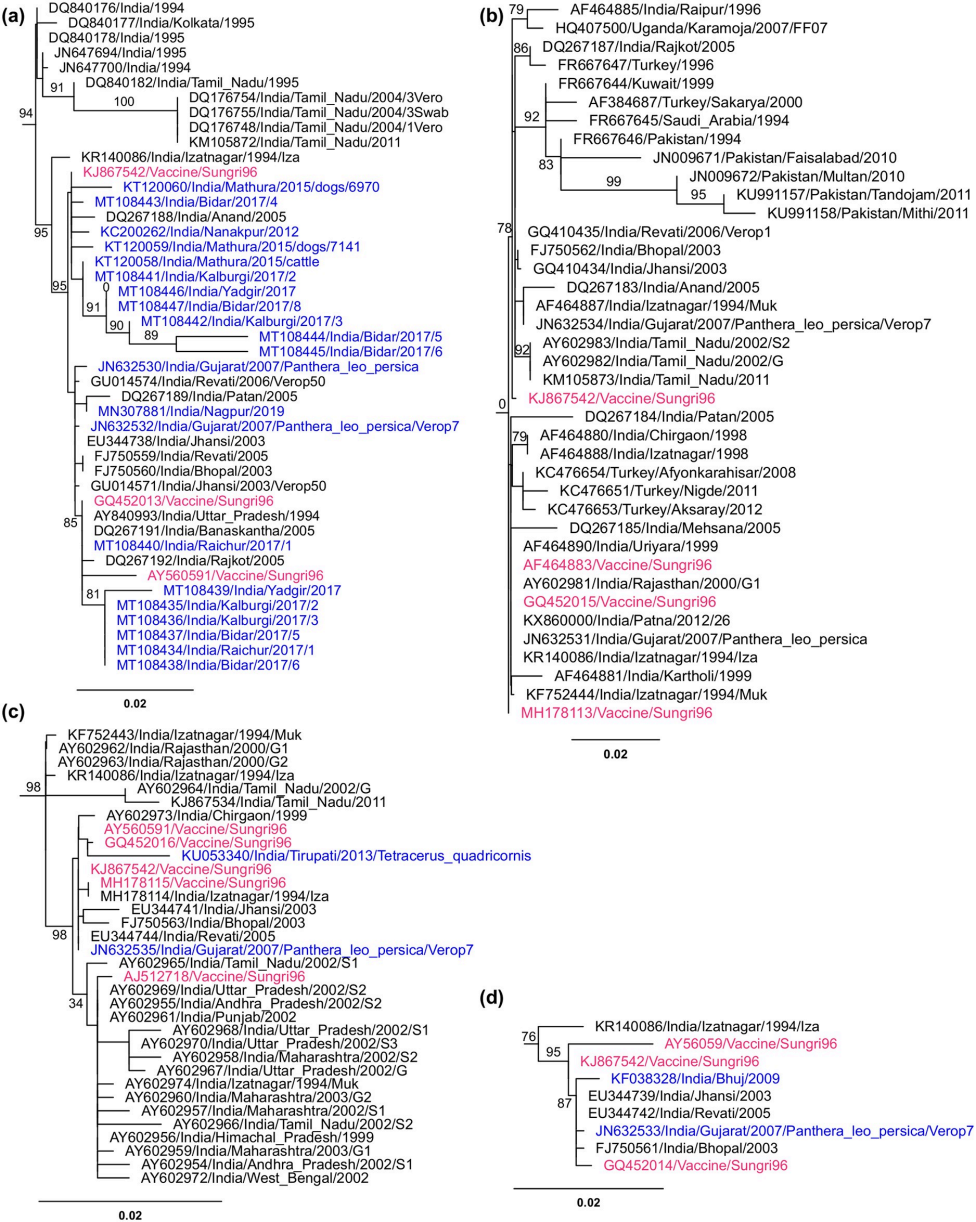
Identification of vaccine/Sungri96 contaminants in the sequence database. Maximum likelihood trees were constructed as described for Fig 3: (a) N gene; (b) F gene; (c) H gene; (d) M gene. For each gene, the clade containing the vaccine/Sungri96 sequences is shown along with SH-aLRT branch support values for relevant branches. Sequences shown in red are those listed in the database as being vaccine/Sungri96, while those in blue are sequences that were excluded due to their unexpected degree of similarity to the vaccine/Sungri96 sequence. The scale bar shows the branch length scale in units of substitutions per site.

Identifying contaminant sequences arising from the Sungri96 vaccine is more difficult, as this vaccine is derived from a more recent isolate and so is inherently less divergent from most of the sequences in the database (of which 94% of the dated samples are from 2000 on) and there are several established isolates from India at around the same date as the vaccine, such as PPRV/India/Izatnagar/1994 (KR140086, KF752443, KF752444). A number of N, M, F and H gene sequences were found that clustered with vaccine/Sungri96 sequences, despite coming from geographically distant parts of India and up to 21 years later in time (Fig 4). For each of N, M and H genes, there was a clear subclade with good support that contained the vaccine/Sungri96 sequences and other Indian sequences from a similar time (late 1990s and early 2000s) and geographical area (Northern India) but also a few sequences from much later (Fig 4), despite most Indian sequences from 2005 onwards forming separate clades. We excluded those vaccine/Sungri96-like sequences from 2006 onwards that grouped with this vaccine; while the observed level of similarity to the vaccine strain is not definitive proof that these sequences are derived from laboratory contamination, there is a strong probability that this is so, and there are sufficient other Indian sequences in the database that it is reasonable to be conservative.

In contrast to the N, M and H gene sequences, the support for the clade containing the known vaccine/Sungri96 sequences in the F gene tree was very low (Fig 4). This clade in the F gene tree was also found to include a number of sequences from outbreaks in Turkey and the Middle East, quite separate from the clades containing other contemporaneous sequences from the same areas. The low statistical support for any of these groupings, and the apparent close relationship of F gene sequences from geographically and temporally distant outbreaks, underlines previous findings that the short F gene region commonly sequenced (positions 237 to 634 in the F gene open reading frame) is not as good as the N gene at separating lineage 4 PPRV isolates [42–44]. We therefore did not exclude any F gene sequences based on their clustering in association with vaccine/Sungri96 F gene sequences. We did exclude the two F gene sequences from a virus supposedly isolated from an Asian lion (JN632531 and JN632534), on the grounds that the N, M and H gene sequences for this isolate are all apparently vaccine/Sungri96, so it is likely that the F gene sequence is as well. The accession numbers of the sequences that are certainly or probably contaminating vaccine virus are listed in the S1 List included in Supporting information.

It should be noted that this kind of laboratory error also happens in the other direction, i.e. sequences stated to come from one of the vaccines were found to be from unrelated strains. KF727981 is supposed to be from the N gene of vaccine/Sungri96 but groups with Iranian and Turkish sequences, while L39878 is supposed to be from the vaccine/Nigeria75 N gene, but groups with Sudanese and Ethiopian viruses from a completely different lineage.

#### (iii) Excess divergence

Sequences were further filtered based on excess divergence, i.e. where the molecular distance from other sequences did not fit with the date of isolation of the virus. Such filtering is commonly applied to large datasets of diverse origin, e.g. [23, 45]. This assumes a single rate of gain of molecular distance, i.e. a simple molecular clock, for all the virus isolates, which is a reasonable approximation for viruses isolated over a relatively restricted temporal range, although it would be an oversimplification for detailed analysis of the ancestral history of PPRV, for example, or its relationship with other viruses [46]. For PPRV sequences, initial TreeTime analysis suggested that it was not possible to assume the same clock applied to all lineages, especially for the F gene sequences, as analysing all F gene sequences together identified 15 of the 18 lineage 2 sequences as “divergent” (not shown). This effect seemed to be due to a difference in molecular clock between lineage 4 and the other lineages, as analysing the sequences after dividing into lineages1+2+3 and lineage 4 prevented this sort of artefact. Divergent sequences were therefore assessed separately for lineages1+2+3 and lineage 4 for H, M, N and F genes and for whole genomes.

**Fig 5:**
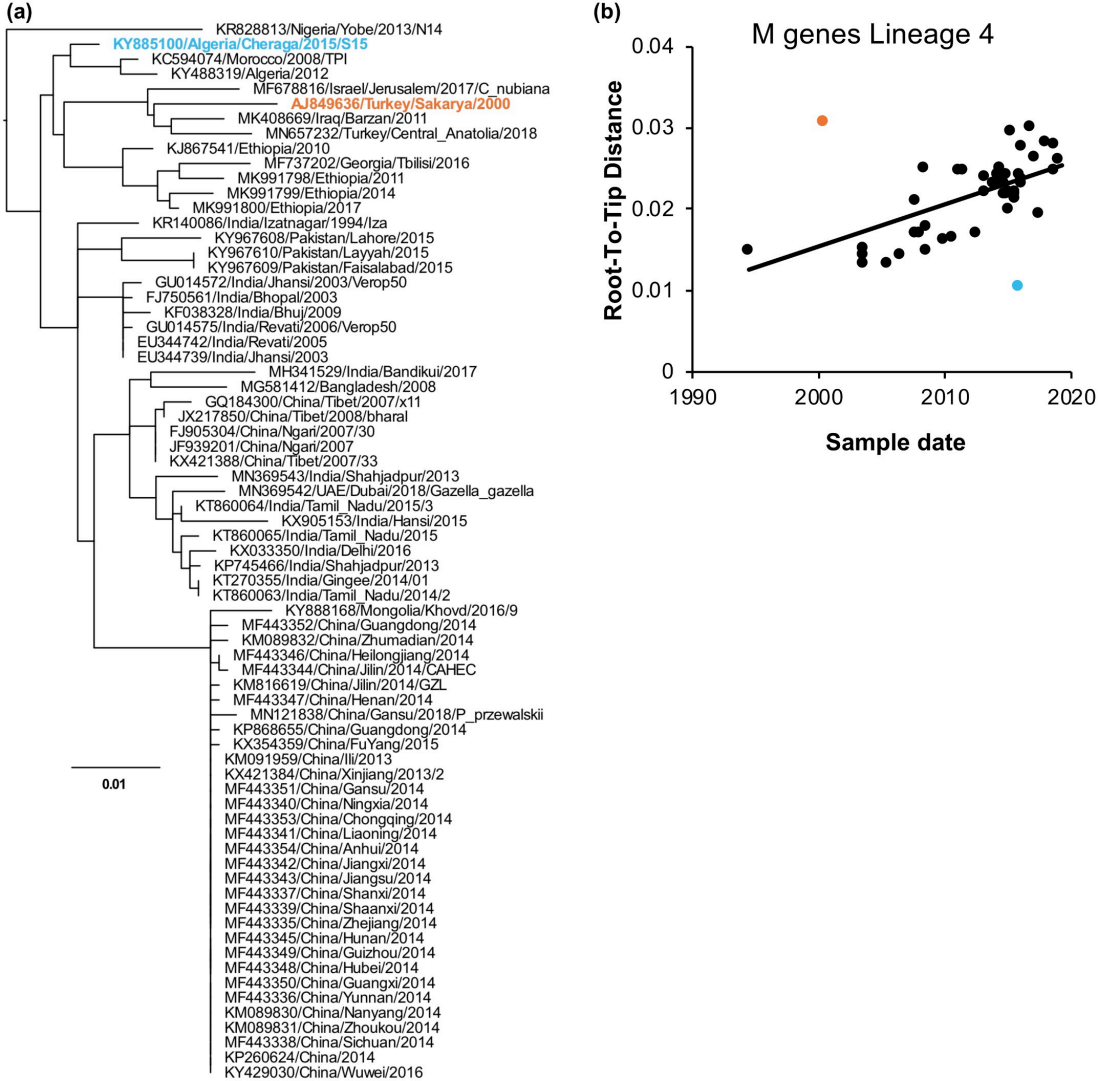
An example of filtering excessively divergent sequences. Sequences were filtered using TreeTime as described in Methods. Shown is the results of this analysis on M gene sequences of lineage 4. (a) Phylogenetic tree containing all sequences at this stage of the filtering process. The tree is rooted at the node which minimises the squared deviations of the sample dates from the calculated date. (b) Plot of the calculated root-to-tip distance for each sequence, given the tree in (a), and the sample date. The straight line represents the strict clock fit to the data. Sequences identified as divergent are highlighted in (a) and (b).

A total of 37 gene sequences and one genome sequence were excluded at this stage, including one partial N gene sequence (KJ124734/Nigeria/Ogun/2013) which was not reliably placed in a specific lineage, being placed in lineage 4 on the best tree found by RAxML, for example, and in lineage 1 on the best tree found by IQ-TREE 2, with low support values in either case. A small example of this divergence filtering (Lineage 4 M gene sequences) is shown in Fig 5, and a larger example in S3 Fig Sequences excluded due to excess divergence are listed in the S1 List. Further information about these isolates (e.g. repeated, preferably longer, sequences, the sequences of further related isolates, or improved date information) will be required to clarify the issues in all these cases.

After these filters had been applied, it was clear that the majority of the sequences in some batches were low quality by one or more of these criteria; we therefore treated those entire batches as suspect and excluded them all (Accession Numbers DQ185576-185591; JX443705-443713; KM659204-659214; MK213753-213757).

We also excluded at this stage the sequences from viruses which were stated to have been passaged a large number of times in cell culture (GU014571-6). The extensive passage in cell culture for these and the vaccine viruses may have introduced unnatural molecular diversity, so that they are no longer properly representative of their parental isolate. The remaining vaccine virus sequences are purely for reference, and should not be included in analyses of phylogenetic evolutionary rates or geographic movement.

### Removal of duplicates

The sequence dataset was then filtered to remove duplicate sequences.

Firstly, we removed duplicate full genomes, i.e. where there were two or more database entries for the same virus isolate. We removed NC_006383 as this is the same strain (PPRV/Turkey/2000), from the same authors, as AJ849636 (the latter was kept as it is the more recent). In the same way, there are three full-length genomes of each of the vaccine/Nigeri75 (HQ197753, KY628761, X74443) and vaccine/Sungri96 (AY560591, KJ867542 and the suspect KF727981) strains. We kept only one of each (KY628761 and KJ867542 respectively), taking the most recent and therefore hopefully the most accurate examples. We also noted that KX421384-7 are four full-length genomes from the same outbreak (China/Xinjiang/2013), and almost completely identical over the whole length of the genome. Only KX421384 was retained in the dataset. That gave 74 complete genome sequences, plus two representing the main vaccine strains. This alignment, as with the gene-specific alignments, is available from the PPR lab network website (https://www.ppr-labs-oie-network.org).

Duplicate removal for individual N, M, F and H genes was done in two stages. First, the separate gene sequences were filtered to remove identical sequences (including situations where one sequence was a subset of another) from the same outbreak, where the repeated sequences add no phylogenetic or phylogeographic information, and may bias analyses simply because some outbreaks are more intensively documented than others. We also removed a group of 16 identical sequences from an outbreak in Chattogram, Bangladesh, in 2018–19 (MN732923-38); these sequences were neither the whole N gene, nor the region of the N gene where the standard PCR is targeted, and from where most of the available sequences are derived. They therefore did not overlap with most of the available sequences, affecting the reliability and stability of phylogenetic analyses.

**Fig 6:**
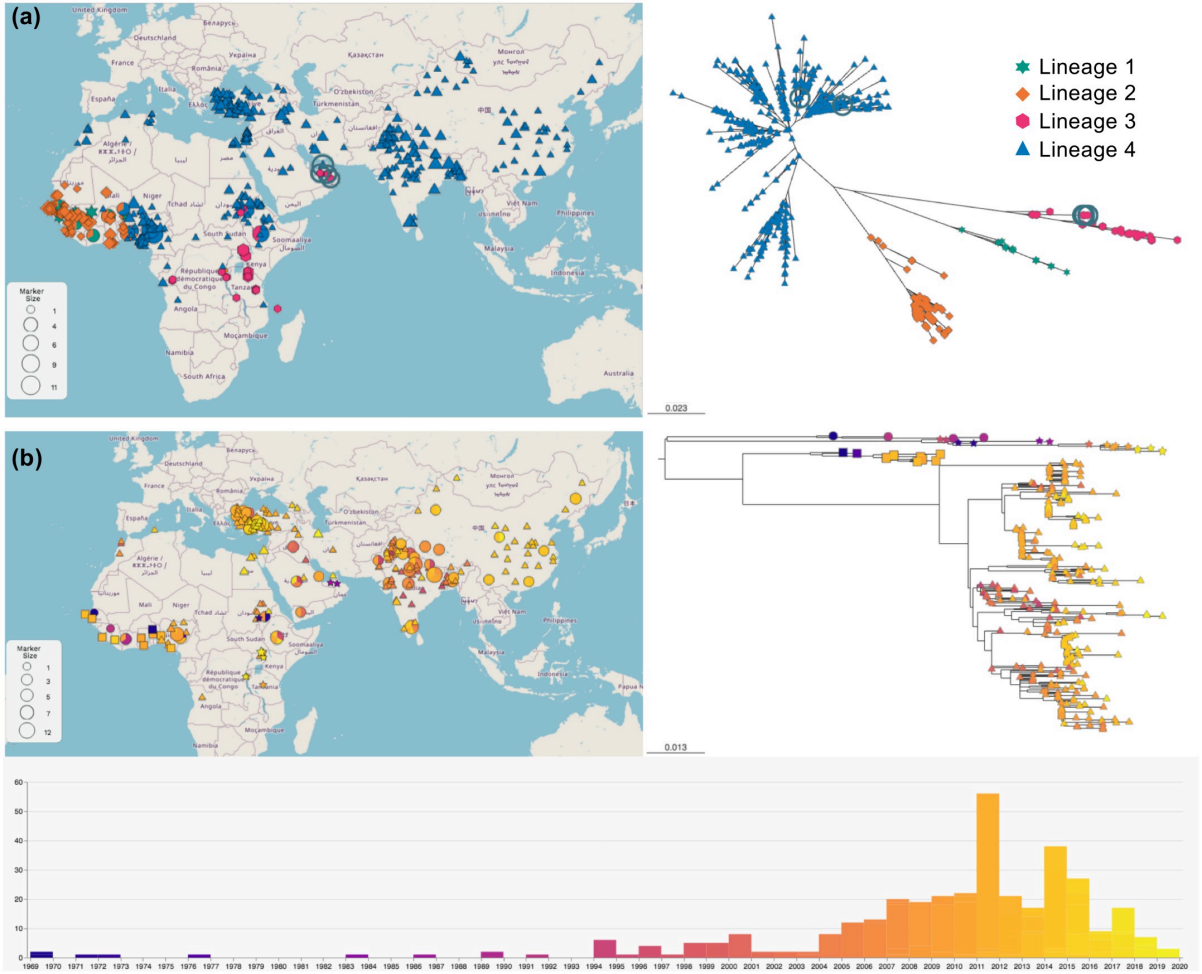
Visualisation of PPRV sequence relationships using MicroReact. (a) Microreact display of the geolocation of unique N gene sequences with associated unrooted tree. Sequences have been coloured by lineage, and the figure shows the selection of a group of Middle Eastern isolates, with simultaneous highlighting of their positions on the tree. (b) Microreact display of the same dataset but using a pseudo-rooted tree and colouring the sequences by sample date; the timeline at the bottom of the figure shows numbers of sequences representing each year and the mapping of dates to colours. Note that there are too many sequences to show sequence names on the respective trees in (a) and (b), but MicroReact displays these names on mouse-over in an interactive display. The maps shown in the figure are public domain maps from the USGS National Map Viewer https://apps.nationalmap.gov/viewer/. Links to the interactive MicroReacts for full PPRV genomes and the N and F gene sequences, plus all underlying metadata, are available from https://www.ppr-labs-oie-network.org/materials-and-protocols/pprv-sequence-datasets.

This process removed 429 sequences, primarily from N and F, leaving 712, 96, 358 and 165 sequences, respectively, in the N, M, F and H gene alignments. The resultant datasets represent quality-filtered sequences which have a unique place/date values, or are from the same place and year but with different sequences; these alignments are tagged “unique”. Such alignments are most useful where variation (or lack of it) is being studied with respect to place as well as time (phylogeography). As an illustration of the utility of this dataset, we have integrated the maximum likelihood tree based on the available unique sequence information with the available geolocation data for each isolate on the web site MicroReact [47]. This allows visualisation of the number and dates of isolates of different lineages on a world map; two example displays are shown in Fig 6. Links to this visualisation tool for all datasets are provided from our web site.

These datasets were further filtered to remove all duplicate sequences, even where the duplicate sequences were from isolates collected in different places or times. This step improves the quality and speed of phylogenetic analysis, and is an important factor in, for example, bootstrap analysis, which assumes that each member of the alignment has a unique sequence. Sequences that are an exact match were identified, and all but one of the identical sequences were removed, keeping the oldest where sequences were from different years. As above, this filtering also identified sequences where a database entry was an exact match to part of a longer entry (a subsequence). Sequences that were subsequences of other database entries were all removed, regardless of date of isolation. This left gene-specific datasets that can be used for purely phylogenetic analyses (“NoDups”). The final numbers for each gene were 519, 74, 228 and 139 for N, M, F and H genes, respectively.

### Dataset pruning

**Fig 7:**
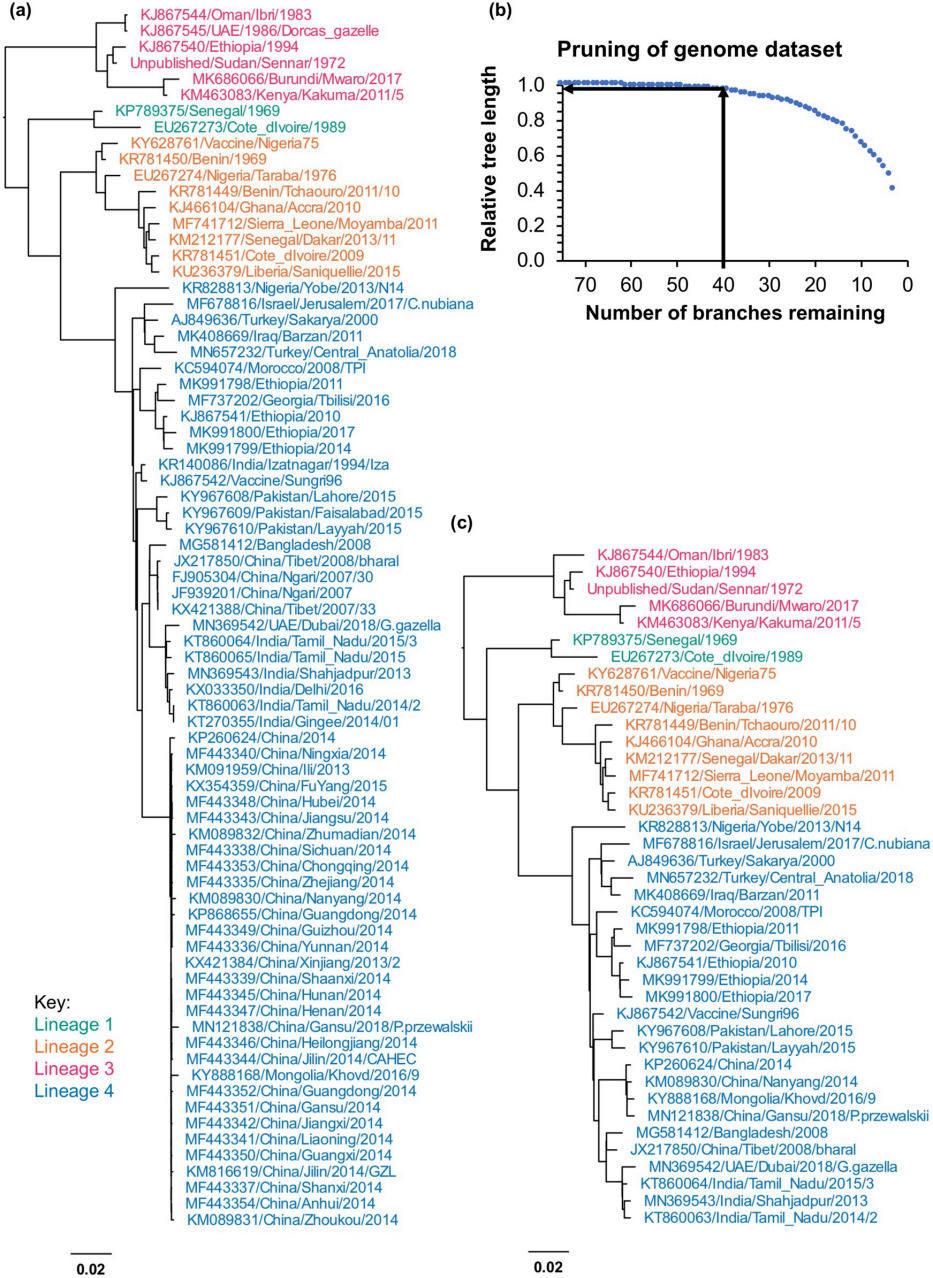
Example of tree pruning to give a simpler dataset. The maximum likelihood tree was calculated for the unique genome sequences and then pruned using *treemmer* to give a simpler but almost equally informative dataset: (a) Full genome sequence tree; (b) relative tree length for the remaining tree at each pruning cycle; (c) maximum likelihood tree calculated from the list of sequences in the pruned tree. (a), (c): Lineage 1 (blue/green), Lineage 2 (orange), Lineage 3 (magenta), Lineage 4 (blue).

These datasets still contain intrinsic bias, in that there are many very similar sequences from some countries, and few from others; in the case of the N and F genes, which are the most commonly sequenced, the data sets are also still very large, even after removing all duplicates. Including all of the available data when analysing new sequences is not usually necessary, nor indeed is it always useful, leading to large and unwieldy phylogenetic trees, as well as being computationally expensive. We reduced each of these datasets to a reasonable minimal dataset using *Treemmer* ([26], which iteratively prunes a tree while retaining as much phylogenetic information (taken as the overall length of the tree branches) as possible; in each cycle it identifies the two closest branches (most similar sequences) and prunes one. As can be seen in Fig 7, even for the current list of 76 full genome sequences, this process provides an objective way to reduce the current bias towards full-length genomes from the China/2013/2014 outbreak while maintaining almost all of the genetic diversity, with 40 genomes (approximately half of the dataset) keeping >95% of the overall tree length (Fig 7b), while the major clades and subclades are all still present (Fig 7c). For the larger sets of individual gene sequences, notably the N and F gene sequences, the benefits of such simplification are even greater, with a subset of 60 N gene or F gene sequences retaining at least half of the full genetic diversity available, even though the number of sequences is reduced to approximately 11% or 24%, respectively (see S4 and S5 Figs). Given the unequal spread of lineages around the world, and the fact that the vast majority of sequences have come from Asian isolates, and therefore lineage 4, we carried out parallel tree pruning with the added constraint that no lineage would go below 8 sequences (see example of F genes in S5 Fig); such datasets may be particularly useful when analysing non-lineage 4 sequences.

## Discussion

Tracking the spread of pathogens and identifying the sources of new outbreaks has more and more relied on the analysis of pathogen genome sequences [48], whether this is a human pathogen such as influenza virus and SARS-CoV2 (e.g. [49, 50]) or one affecting livestock such as foot-and-mouth disease (e.g. [51]). In all cases, the value of such analyses depends entirely on the quality of the sequence information used. Our experience in curating the available sequence information for PPRV suggests that some care must be taken in selecting sequence data for use in analyses, as a significant fraction of the available data (207 out of 1762 available non-vaccine N,M,F,H or genome sequences) was found to be suspect. Some previous analyses (e.g. [8–10, 52]) may benefit from repeating with the datasets provided here.

Our analyses of the available sequence data has highlighted several points. Firstly, it is important for all laboratories contributing PPRV sequence data to the international repositories (GenBank/European Nucleotide Archive/DNA Databank of Japan) to take simple steps to improve, or at least control, the quality of the data submitted. Such steps should always include:

1. Checking that any new sequence obtained aligns with existing data without insertion of gaps in either the existing or the new sequences in order to achieve alignment, and reviewing raw trace data where necessary; insertions/deletions are not unknown in morbilliviruses, but those observed to date have been confined to less conserved untranslated regions, and have been in multiples of six bases.
2. Ensuring that the open reading frame of the viral proteins is maintained, again reviewing raw trace data as required.
3. Recognising when the sequence obtained from a PCR product is an unexpectedly close match to a virus (usually a vaccine strain) being used as a PCR control in the laboratory, or to any other virus isolate being used in the laboratory, and repeating the work where necessary.
4. Not including sequence data where the quality parameter provided by the sequencer is low (frequently a problem at the end of sequencing reads).
5. Always trimming the PCR primer sequence away from new sequences before further analysis and upload to the public database.

These simple steps may prevent errors creeping into the literature. It was observed in the work presented here that, among the sequences which are essentially identical to the vaccine/Sungri96 virus were several that have been reported to have been “isolated” from dogs and cattle in 2015 [53], from a wild bovid in 2013 [54] or from an Asiatic lion in 2007 [55]. In each of these cases the viruses circulating in sheep and goats in those areas and times belonged to other clades. Similarly, finding the sequence of the vaccine/Nigeria75 strain in an animal in China led to the claim that this vaccine, used without incident in tens of millions of animals all over Asia and Africa for 40 years, had “reverted to virulence” [56], while the possibility of laboratory contamination was not considered [57]. Note that, in the cases of clear PPR-like disease, such as the captive antelope in India [54] or the water deer in China [56], the finding of vaccine sequence does not mean the animal was not infected with wild type PPRV, just that the laboratory test has failed to detect the actual infectious agent.

In addition to filtering for poor sequence quality by checking for extraneous gaps and stop codons, we also filtered by divergence. This method has the advantage that it can be applied objectively and incorporated into a pipeline for processing new data, as has been implemented, for example, in the NextStrain monitoring site [45]. It should be emphasised that, while this method is most useful for the 94% of sequences that represent recent isolates (from the year 2000 onwards), the small number of virus sequences we have representing older isolates makes estimating the molecular clock for this period difficult, nor is it likely that a strict molecular clock applies over a longer period of time. In applying this filter to the available PPRV data, it drew attention not only to obvious outliers with very long branches, such as MN496449/Sierra_Leone/Kenema/2018/38 (S3 Fig), but also those which had anomalously short branch lengths, such as several sequences from Nigeria in 2012 and 2013, which were not related either to each other, nor any contemporaneous sequences from the same region (S3 Fig). Until these isolates are resequenced, it is not possible to say if these divergent sequences represent laboratory errors or new, highly divergent strains of the virus. The generally low rate of sequence divergence among PPRV strains that we have observed in the data collected here means that it is reasonable at this stage to exclude these sequences, pending further information.

The generally low rate of sequence divergence also means that, for phylogenetic analyses beyond simple lineage identification, longer sequences are now necessary. As noted above, and by others [42, 43], the standard short N gene sequence seems better than the standard short F gene sequence in distinguishing subclades of PPRV in lineage 4. However, we have also found cases where different viruses are poorly distinguished by the N gene sequence. For example, the standard short N gene sequences of Sudan/Sennar/1972, Ethiopia/1994 and two isolates from Sudan in 2000 (HQ131946 and HQ131919) are all identical, with only a few differences seen between Sudan/1972 and Ethiopia/1994 in the whole N gene; in contrast, the short F gene sequences clearly distinguished them. F gene sequences may therefore be helpful in analyses of viruses of lineage 3, which is still circulating in East Africa.

The other major problem we found in assembling these data was the quality of metadata in many database entries. Detailed information on sample collection date is rarely provided, indeed a few entries did not even record the year of isolation (AF344886, AY948429, FR696359, FR714844 and MH999830). While further information on the date and location of sample collection could be found through literature searches, it would be of great benefit to further research if this information is uploaded to the data repositories. Specific location information (co-ordinates) are also of great benefit for those countries where local names are either not yet recorded in global databases (as is true of large parts of Africa) or where the need to transcribe between different alphabets (e.g. Arabic to Roman) can lead to multiple different forms for a local name; for example, the town in Sudan where one of the two earliest-identified PPRV isolates was isolated [58] was recorded in that paper as Mieliq, but has also been recorded as Mielik and Mielig, and is now known (on Geonames and OpenStreetMap) as Al Mu’ayliq, with alternative spellings Elmielg, El Mi’eiliq, El Mieliq, Maleig and Mieliq. Clarifying the metadata submitted to the database by providing the co-ordinates of either the location (if the person collecting the sample had access to a GPS) or even the map reference of the nearest town or village, would prevent confusion. For the existing data, we have identified the smallest geographic unit (village, town, state or country) compatible with the published data, and used the centroid of that as the location for the purposes of the dataset. This information is available along with the curated sequence alignments.

As outlined in Methods, we have adopted a standardised naming system for PPRV isolates and sequences. While there is no internationally-agreed standard, we would like to encourage other scientists and epidemiologists to consider using this system as a harmonised naming convention for PPRV: it has the advantage of being relatively easy for either humans or computers to parse for country and date information, and is certainly more generally informative than using the originating laboratory code, such as “PK-SM16-N_PPR_ICT”, “CAD485/18_2_PPR_Leh” or “7_OD/Gan/2016”. GenBank entries can accommodate both the harmonised name (as “Isolate” or “Strain” and the laboratory code (as “Specimen_voucher”). While we have only included the host type in the name where this was non-typical, i.e. something other than domestic sheep or goats, it is of course open to others to include this information for all types of hosts; as long as it occupies the same position in the harmonised name, it will not affect computerised parsing of the PPRV identity.

Our collation of the available data showed a large amount of sequence duplication in the sequence repository. In addition to cases where multiple samples were sequenced from the same outbreak location, there were several examples where it was clear that the same samples had been sequenced, and the sequences uploaded to the database, twice. In other cases, laboratories had improved the length of sequence data for a specific gene from a specific sample and, instead of editing the existing database entry, had simply uploaded the longer sequence, meaning one sample could give rise to two or three sequences in the database. This can lead to confusion through the assumption that all of these sequences are separate samples. By searching for duplicate samples and duplicate entries, we have removed this level of bias in our datasets.

We also detected clear evidence of mixed genome sequences in at least 6 full-length genome sequences in the database. While the “parent” viruses in each case are geographically and temporally distinct, making it extremely unlikely that these are the sequences of actual recombinants that arose in nature, we cannot completely discard the possibility that virus recombination events did actually happened during accidental or planned co-infections in cell cultures in the laboratory. Whether they arose due to accident or through actual recombination, these sequences should not be included in phylogenetic analyses.

In addition to curating sets of sequence data with, we hope, most of the erroneous sequences removed along with duplication-induced bias, we have demonstrated here that the available data can be simplified without great loss of information by reducing the set of sequences used for analyses. For lineage identification, an unknown sequence can be compared with a 60 sequence subset of sequences from the relevant gene, making for more rapid analysis and a clearer result. Similar reductions in size could be made with constraints on a minimum number of sequences for each country, for example, depending on the requirements of the analysis. The curated datasets described here, both the “Unique” and “NoDups” alignments and the streamlined datasets, are available from the website of the PPRV diagnostic laboratory network (https://www.ppr-labs-oie-network.org/materials-and-protocols/pprv-sequence-datasets) and will be updated regularly.

## Supporting information

S1 Fig Sequence screening and filtering workflow. (PDF)

S2 Fig Alignment problems in the M-F untranslated region of PPRV genomes. (PDF)

S3 Fig An example of filtering excessively divergent sequences. (PDF)

S4 Fig Tree pruning to give a simpler dataset of N gene sequences. (PDF)

S5 Fig Tree pruning to give a simpler dataset of F gene sequences. (PDF)

S1 Table Corrections made to full PPRV genome sequences in the database. (PDF)

S1 List Text file listing all excluded sequences by accession number and cause. (TXT)
