# Supplementary material for: A curated dataset of peste des petits ruminants virus sequences for molecular epidemiological analyses
Source: PLoS One. 2022 Feb 10;17(2):e0263616. doi: 10.1371/journal.pone.0263616 (PMC8830648; doi:10.1371/journal.pone.0263616)
Supplement: S1 Fig — (PDF) [file pone.0263616.s001.pdf]

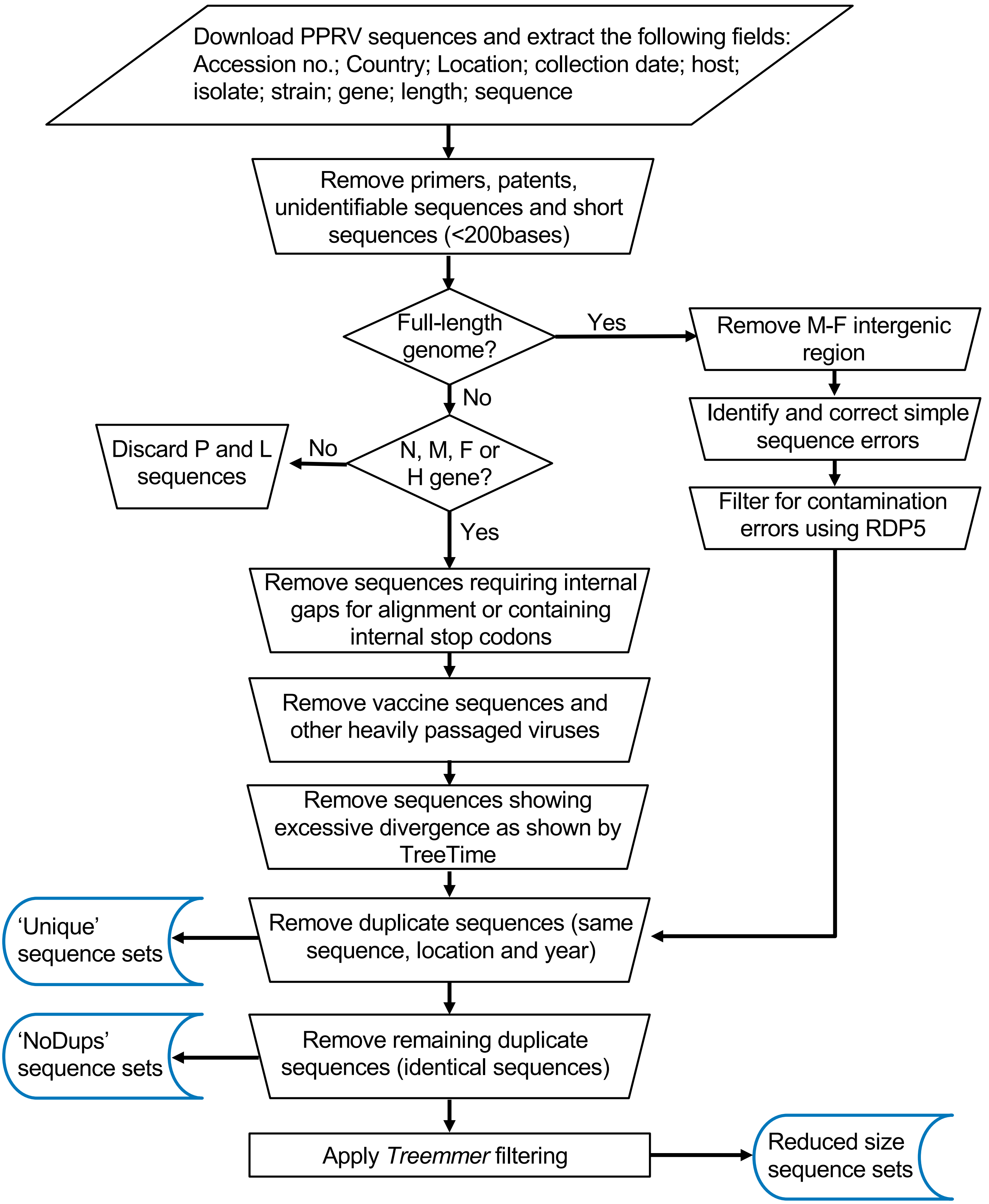

S1 Fig Sequence screening and filtering workflow

The various stages of filtering applied to the collection of PPRV sequences in the database is shown in this figure. The final output datasets are shown as Saved Data shapes (blue).
