# Supplementary material for: A curated dataset of peste des petits ruminants virus sequences for molecular epidemiological analyses
Source: PLoS One. 2022 Feb 10;17(2):e0263616. doi: 10.1371/journal.pone.0263616 (PMC8830648; doi:10.1371/journal.pone.0263616)
Supplement: S2 Fig — (PDF) [file pone.0263616.s002.pdf]

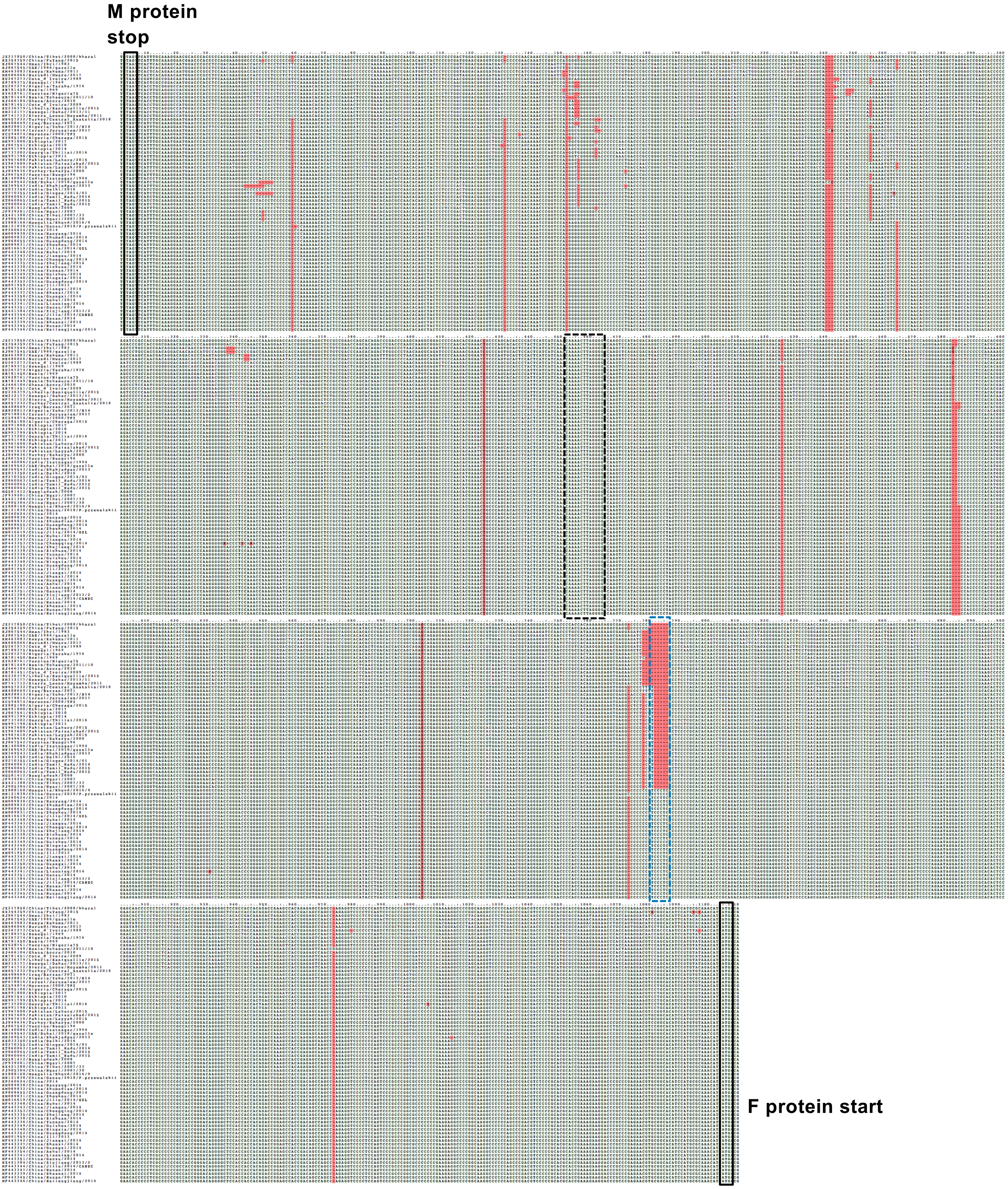

S2 Fig Alignment problems in M-F untranslated region of PPRV genomes
The available PPRV genome sequences were aligned with MAFFT under constraints to penalise gap opening unless it provided strong benefit to the alignment score. The region between the end of the M open reading frame and the start of the F open reading frame (respective stop and start codons boxed) was extracted and shown here to illustrate the extensive gapping (highlighted in red) that is required to maintain alignment between sequences. The known introduction of 6 extra bases in a lineage of PPRV originally identified in China is highlighted (blue dashed line). The intergenic stop transcription/start transcription site is also boxed (dashed line).
