# Supplementary material for: A curated dataset of peste des petits ruminants virus sequences for molecular epidemiological analyses
Source: PLoS One. 2022 Feb 10;17(2):e0263616. doi: 10.1371/journal.pone.0263616 (PMC8830648; doi:10.1371/journal.pone.0263616)
Supplement: S3 Fig — (PDF) [file pone.0263616.s003.pdf]

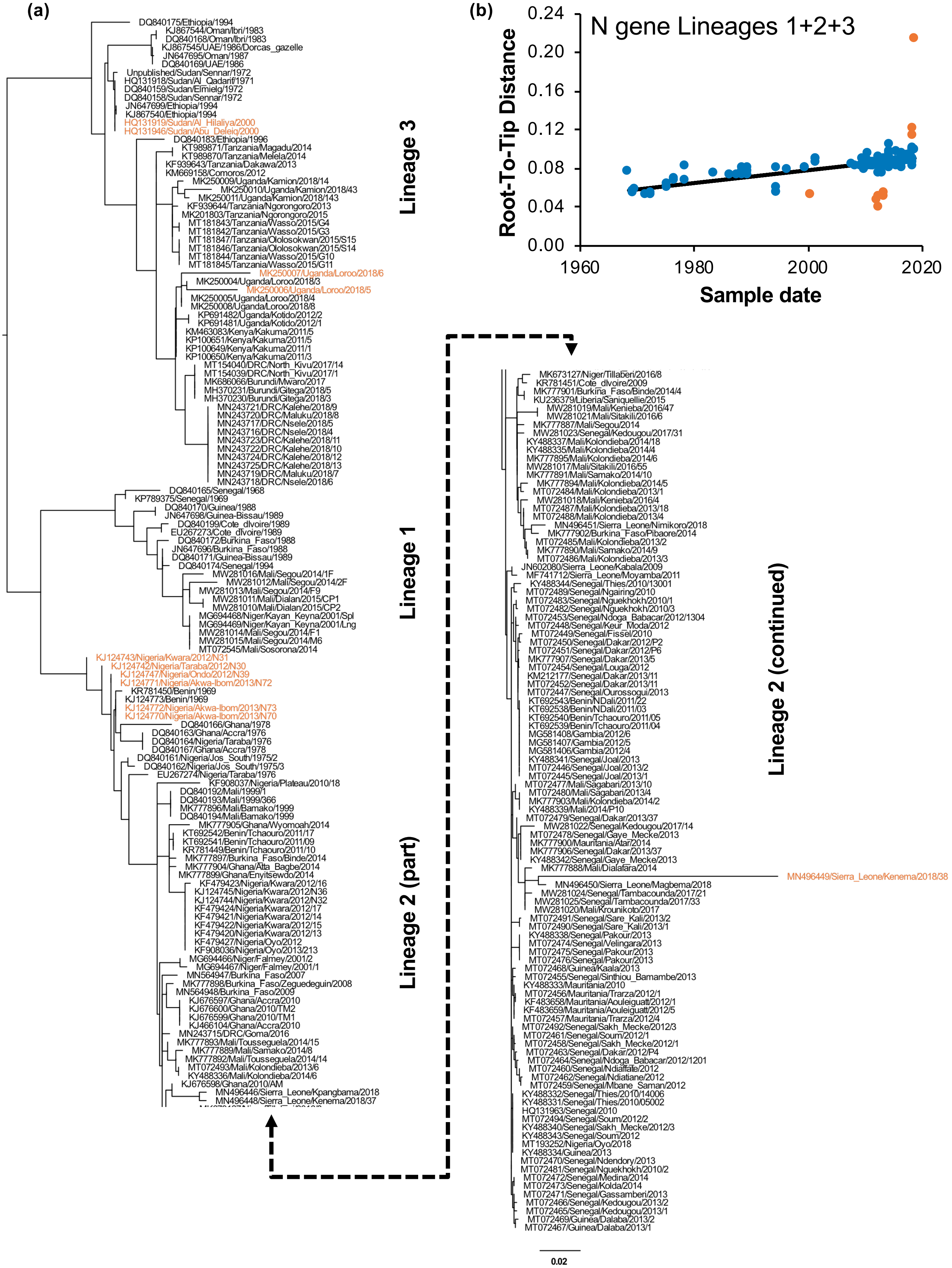

S3 Fig. An example of filtering excessively divergent sequences. Sequences were filtered using TreeTime as described in Methods. Shown is the results of this analysis on N gene sequences of lineages 1,2 and 3. (a) Phylogenetic tree containing all sequences at this stage of the filtering process. The tree is rooted at the node which minimises the squared deviations of the actual sample dates from the calculated date, and has been split in order to fit in the figure. (b) Plot of the calculated root-to-tip distance for each sequence, given the tree in (a), and the sample date. The straight line represents the strict clock fit to the data. Sequences identified as divergent are highlighted in (a) and (b).
