# Supplementary material for: A curated dataset of peste des petits ruminants virus sequences for molecular epidemiological analyses
Source: PLoS One. 2022 Feb 10;17(2):e0263616. doi: 10.1371/journal.pone.0263616 (PMC8830648; doi:10.1371/journal.pone.0263616)
Supplement: S4 Fig — (PDF) [file pone.0263616.s004.pdf]

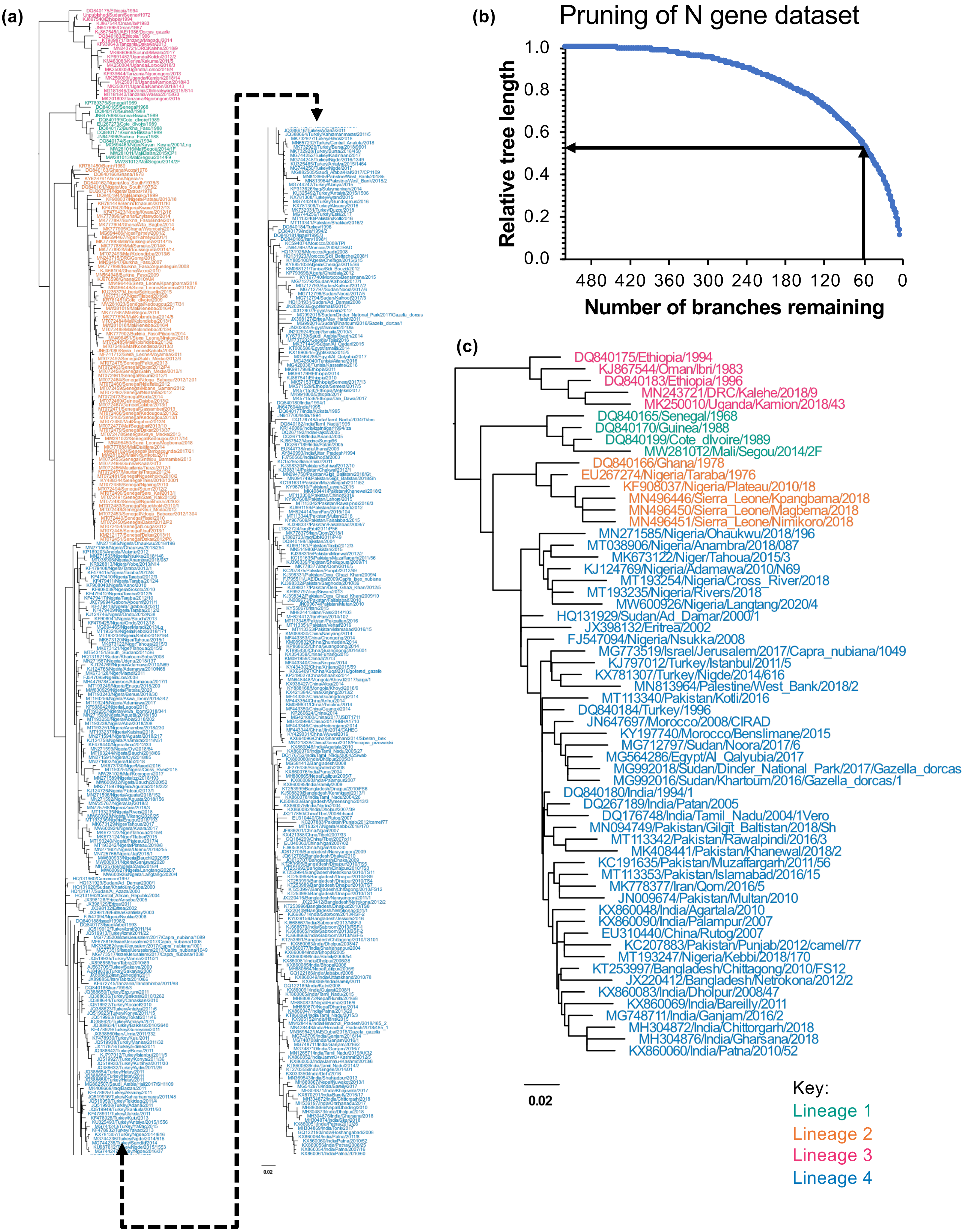


**S4 Fig. Tree pruning to give a simpler dataset of N gene sequences.** The maximum likelihood tree was calculated for the unique N gene sequences and then pruned to give a simpler but equally informative dataset: (a) Full N gene sequence tree (split); (b) plot showing relationship between tree length and degree of pruning; (c) Maximum likelihood tree based on pruned list of 60 sequences. (a), (c): Lineage 1, Lineage 2, Lineage 3, Lineage 4
