# Supplementary material for: A curated dataset of peste des petits ruminants virus sequences for molecular epidemiological analyses
Source: PLoS One. 2022 Feb 10;17(2):e0263616. doi: 10.1371/journal.pone.0263616 (PMC8830648; doi:10.1371/journal.pone.0263616)
Supplement: S5 Fig — (PDF) [file pone.0263616.s005.pdf]

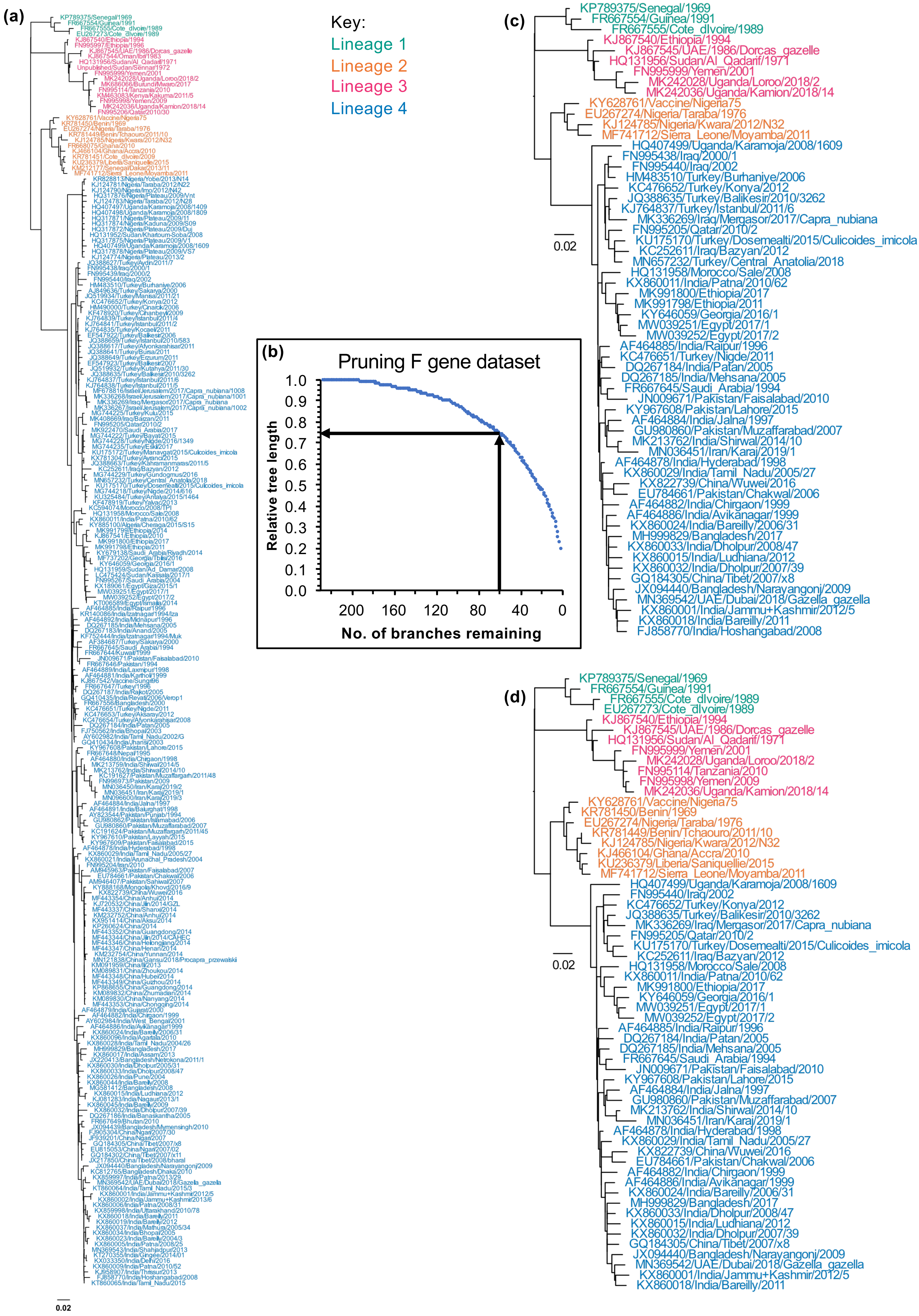

(a)
0.02
Key:
Lineage 1
Lineage 2
Lineage 3
Lineage 4
(b)
Pruning F gene dataset
Relative tree length
No. of branches remaining
1.0
0.9
0.8
0.7
0.6
0.5
0.4
0.3
0.2
0.1
0.0
200
160
120
80
40
0
(c)
KP789375/Senegal/1969
FR667554/Guinea/1991
FR667555/Cote_dIvoire/1989
KJ867540/Ethiopia/1994
KJ867545/UAE/1986/Dorcas_gazelle
HQ131956/Sudan/Al_Qadarif/1971
FN995999/Yemen/2001
MK242028/Uganda/Loroo/2018/2
MK242036/Uganda/Kamion/2018/14
KY628761/Vaccine/Nigeria75
EU267274/Nigeria/Taraba/1976
KJ124785/Nigeria/Kwara/2012/N32
MF741712/Sierra_Leone/Moyamba/2011
HQ407499/Uganda/Karamoja/2008/1609
FN995438/Iraq/2000/1
FN995440/Iraq/2002
HM483510/Turkey/Burhaniye/2006
KC476652/Turkey/Konya/2012
JQ388635/Turkey/Balikesir/2010/3262
KJ764837/Turkey/Istanbul/2011/6
MK336269/Iraq/Mergasor/2017/Capra_nubiana
FN995205/Qatar/2010/2
KU175170/Turkey/Dosemealti/2015/Culicoides_imicola
KC252611/Iraq/Bazyan/2012
MN657232/Turkey/Central_Anatolia/2018
HQ131958/Morocco/Sale/2008
KX860011/India/Patna/2010/62
MK991800/Ethiopia/2017
MK991798/Ethiopia/2011
KY646059/Georgia/2016/1
MW039251/Egypt/2017/1
MW039252/Egypt/2017/2
AF464885/India/Raipur/1996
KC476651/Turkey/Nigde/2011
DQ267184/India/Patan/2005
DQ267185/India/Mehsana/2005
FR667645/Saudi_Arabia/1994
JN009671/Pakistan/Faisalabad/2010
KY967608/Pakistan/Lahore/2015
AF464884/India/Jalna/1997
GU980860/Pakistan/Muzaffarabad/2007
MK213762/India/Shirwal/2014/10
MN036451/Iran/Karaj/2019/1
AF464878/India/Hyderabad/1998
KX860029/India/Tamil_Nadu/2005/27
KX822739/China/Wuwei/2016
EU784661/Pakistan/Chakwal/2006
AF464882/India/Chirgaon/1999
AF464886/India/Avikanagar/1999
KX860024/India/Bareilly/2006/31
MH999829/Bangladesh/2017
KX860033/India/Dholpur/2008/47
KX860015/India/Ludhiana/2012
KX860032/India/Dholpur/2007/39
GQ184305/China/Tibet/2007/x8
JX094440/Bangladesh/Narayangonj/2009
MN369542/UAE/Dubai/2018/Gazella_gazella
KX860001/India/Jammu+Kashmir/2012/5
KX860018/India/Bareilly/2011
FJ858770/India/Hoshangabad/2008
0.02
(d)
KP789375/Senegal/1969
FR667554/Guinea/1991
FR667555/Cote_dIvoire/1989
EU267273/Cote_dIvoire/1989
KJ867540/Ethiopia/1994
KJ867545/UAE/1986/Dorcas_gazelle
HQ131956/Sudan/Al_Qadarif/1971
FN995999/Yemen/2001
MK242028/Uganda/Loroo/2018/2
FN995114/Tanzania/2010
FN995998/Yemen/2009
MK242036/Uganda/Kamion/2018/14
KY628761/Vaccine/Nigeria75
KR781450/Benin/1969
EU267274/Nigeria/Taraba/1976
KR781449/Benin/Tchaouro/2011/10
KJ124785/Nigeria/Kwara/2012/N32
KJ466104/Ghana/Accra/2010
KU236379/Liberia/Saniquellie/2015
MF741712/Sierra_Leone/Moyamba/2011
HQ407499/Uganda/Karamoja/2008/1609
FN995440/Iraq/2002
KC476652/Turkey/Konya/2012
JQ388635/Turkey/Balikesir/2010/3262
MK336269/Iraq/Mergasor/2017/Capra_nubiana
FN995205/Qatar/2010/2
KU175170/Turkey/Dosemealti/2015/Culicoides_imicola
KC252611/Iraq/Bazyan/2012
HQ131958/Morocco/Sale/2008
KX860011/India/Patna/2010/62
MK991800/Ethiopia/2017
KY646059/Georgia/2016/1
MW039251/Egypt/2017/1
MW039252/Egypt/2017/2
AF464885/India/Raipur/1996
DQ267184/India/Patan/2005
DQ267185/India/Mehsana/2005
FR667645/Saudi_Arabia/1994
JN009671/Pakistan/Faisalabad/2010
KY967608/Pakistan/Lahore/2015
AF464884/India/Jalna/1997
GU980860/Pakistan/Muzaffarabad/2007
MK213762/India/Shirwal/2014/10
MN036451/Iran/Karaj/2019/1
AF464878/India/Hyderabad/1998
KX860029/India/Tamil_Nadu/2005/27
KX822739/China/Wuwei/2016
EU784661/Pakistan/Chakwal/2006
AF464882/India/Chirgaon/1999
AF464886/India/Avikanagar/1999
KX860024/India/Bareilly/2006/31
MH999829/Bangladesh/2017
KX860033/India/Dholpur/2008/47
KX860015/India/Ludhiana/2012
KX860032/India/Dholpur/2007/39
GQ184305/China/Tibet/2007/x8
JX094440/Bangladesh/Narayangonj/2009
MN369542/UAE/Dubai/2018/Gazella_gazella
KX860001/India/Jammu+Kashmir/2012/5
KX860018/India/Bareilly/2011
0.02

S5 Fig. Tree pruning to give a simpler sets of F gene sequences. The maximum likelihood tree was calculated for the "NoDups" set of F gene sequences and then pruned to give a simpler but almost equally informative dataset: (a) Full F gene sequence tree; (b) plot showing how relative tree length decreases during pruning; (c) pruned tree without lineage conservation; (c) pruned tree with lineage conservation. Note that in (d) the two vaccine strains were deliberately retained by assigning them to their own "lineage". (a), (d), (d): Lineage 1, Lineage 2, Lineage 3, Lineage 4
