# Supplementary material for: A curated dataset of peste des petits ruminants virus sequences for molecular epidemiological analyses
Source: PLoS One. 2022 Feb 10;17(2):e0263616. doi: 10.1371/journal.pone.0263616 (PMC8830648; doi:10.1371/journal.pone.0263616)
Supplement: S1 Table — (PDF) [file pone.0263616.s006.pdf]

S1 Table. Corrections made to full PPRV genome sequences in the database

Note that, in addition to these specific corrections, many sequences would have required additional gaps in the region between 4400 and 5500 to maintain the alignment (S1 Fig).

Sequence accession number	Full isolate name	Error position in original sequence	Correction made
KM089831	China/Zhumadian/2014	2498	Remove “GGG” left from P mRNA editing
KM816619	China/Jilin/2014/GZL	4754	Remove 2 repeats of bases 4702-4753 (104 bases total)
KP868655	China/Guangdong/2014	2498	Remove “G” left from P mRNA editing
KP868655	China/Guangdong/2014	2427	Insert gap to maintain reading frame
MN369542	India/Shahjadpur/2013	15939	Remove “CAAGCT” not present in any other sequence
